# Comparison of Local Information Indices Applied in Resting State Functional Brain Network Connectivity Prediction

**DOI:** 10.3389/fnins.2016.00585

**Published:** 2016-12-27

**Authors:** Chen Cheng, Junjie Chen, Xiaohua Cao, Hao Guo

**Affiliations:** ^1^Department of Computer Science and Technology, Taiyuan University of TechnologyTaiyuan, China; ^2^National Laboratory of Pattern Recognition, Institute of Automation, Chinese Academy of SciencesBeijing, China; ^3^Department of Psychiatry, First Hospital of Shanxi Medical UniversityTaiyuan, China

**Keywords:** functional connectivity, local information, link prediction, brain network, graph theory

## Abstract

Anatomical distance has been widely used to predict functional connectivity because of the potential relationship between structural connectivity and functional connectivity. The basic implicit assumption of this method is “distance penalization.” But studies have shown that one-parameter model (anatomical distance) cannot account for the small-worldness, modularity, and degree distribution of normal human brain functional networks. Two local information indices–common neighbor (CN) and preferential attachment index (PA), are introduced into the prediction model as another parameter to emulate many key topological of brain functional networks in the previous study. In addition to these two indices, many other local information indices can be chosen for investigation. Different indices evaluate local similarity from different perspectives. Currently, we still have no idea about how to select local information indices to achieve higher predicted accuracy of functional connectivity. Here, seven local information indices are chosen, including CN, hub depressed index (HDI), hub promoted index (HPI), Leicht-Holme-Newman index (LHN-I), Sørensen index (SI), PA, and resource allocation index (RA). Statistical analyses were performed on eight network topological properties to evaluate the predictions. Analysis shows that different prediction models have different performances in terms of simulating topological properties and most of the predicted network properties are close to the real data. There are four topological properties whose average relative error is less than 5%, including characteristic path length, clustering coefficient, global efficiency, and local efficiency. CN model shows the most accurate predictions. Statistical analysis reveals that five properties within the CN-predicted network do not differ significantly from the real data (*P* > 0.05, false-discovery rate method corrected for seven comparisons). PA model shows the worst prediction performance which was first applied in models of growth networks. Our results suggest that PA is not suitable for predicting connectivity in a small-world network. Furthermore, in order to evaluate the predictions rapidly, prediction power was proposed as an evaluation metric. The current study compares the predictions of functional connectivity with seven local information indices and provides a reference of method selection for construction of prediction models.

## Introduction

As a combination of non-invasive brain imaging techniques and complex network theory, brain networks have been widely used to characterize the morphology (Hagmann et al., 2008) and functional characteristics (Buldyrev et al., 2010) of human brain. Recently, increasing number of researchers have focused on the relationship between structural and functional networks. Structural connectivity has been defined with diffusion imaging and tractography, while functional connectivity has been defined as a time-series correlation between regions of interest (ROIs). Studies have found out structural connectivity is closely related to resting-state functional connectivity at both macro- (Honey et al., 2009; Hermundst et al., 2013) and micro-scales (Wang et al., 2013). Related studies have demonstrated that regions with structural connectivity also exhibit a strong functional connectivity. This finding suggests that functional connectivity might be predicted by characteristics of brain topology (Alexander-Bloch et al., 2013b; Ercsey-Ravasz et al., 2013). Here, “predict” means to deduce the strength or existence of functional connectivity between two ROIs in a resting-state network. The prediction model is mathematical expression of the prediction method.

On the contrary, some studies have shown that strong functional connectivity may exist among regions lacking structural connectivity (Honey et al., 2009). This suggests that information in the network is not only transmitted directly through structural paths, but may also be affected by network topology (Adachi et al., 2012). Additionally, analyses of the anatomical distance (Euclidean distance) between brain regions have shown that functional connectivity can be interpreted as a “distance penalty.” That means the closer two brain regions are, the stronger the functional connectivity is (Alexander-Bloch et al., 2013a,b).

However, long-distance functional interactions cannot be explained by the distance penalty (Vértes et al., 2012), implying that relying solely on anatomical distance is not enough and we need to combine other factors to achieve better predictions of resting-state brain functional connectivity. Some researchers regard neuronal activity as a bridge between structural and functional connectivity. Several models of neural activity have been proposed, including neural mass models (Ponten et al., 2010), neural field models (Power et al., 2013), the Kuramoto model (Cabral et al., 2011), and spiking models (Nakagawa et al., 2013). Other researchers have applied brain network topological information as parameters to model functional connectivity. Nodal degree is a commonly used attribute based on the basic assumptions of random models. The probability of a connection existing between the two regions is proportional to the product of their degrees (Newman, 2010). Several different network topological properties have been proposed to predict the existence of connectivity in resting-state functional brain networks, including structural degree (Tewarie et al., 2014), degree distribution (Friedman et al., 2014), network communication measure (Goñi et al., 2014), and local information (Vértes et al., 2012).

Local information is the simplest direct method in link-prediction research, which utilizes relevant network topological information to predict the possibility of an edge between two given nodes in a network (Getoor and Diehl, 2005). Link prediction reflects the effect of inherent network topology characteristics during the process of network evolution (Wang et al., 2012). As a measurement of topological similarity between two given nodes, local information is the most commonly used method to predict the probability of connections between them (Lü and Zhou, 2011). Because of its significant practical value, local information has been widely used in several scientific fields including information research (Popescul and Ungar, 2003), biomedical research (Stumpf et al., 2008), mobile communications (Dasgupta et al., 2008), and social networks (Kossinets, 2006; Kumar et al., 2010). In neuroscience, methods that use local information have been applied to simulate neural remodeling that occurs during the learning and memorizing tasks (Ziv and Ahissar, 2009), predicting connectivity of neuronal synapses in the rat primary visual cortex (Bock et al., 2011), optimizing component rearrangements to reduce total wiring length in the macaque nervous system (Kaiser and Hilgetag, 2006), analyzing network properties in the *Caenorhabditis.elegans* neuronal network (Varshney et al., 2011), and constructing local neuronal circuits in patients with autism (Markram et al., 2007).

Although neuroscience investigations that apply local information methods have been conducted at the micro-scale, few have done macro-scale analyses. Vértes applied local information methods to connectivity prediction in resting-state functional brain networks and showed that the best predictions came from the model that combined anatomical distance with the indices—“common neighbor” (CN) (Vértes et al., 2012) among a dozen models. Common neighbor is one of local information indices whose mathematical definition is the number of neighbors that two locations x and y have in common.

Local information reveals the topological similarity of nodes and reflects local topological coherence in networks (Lü et al., 2015). The basic implicit assumption of local information is that the more similar the topology between two given nodes, the higher the probability of an edge existing between them (Lü and Zhou, 2011). This method has been validated by the research in which two local information indices—“common neighbor” and “preferential attachment” (PA)—were introduced with the mathematical definition of the models for predicting resting-state functional connectivity (Vértes et al., 2012). In addition to these two indices, many other local information indices can be chosen for investigation. Different indices evaluate nodal similarity from different perspectives. Currently, we still have no idea about how to select a local information index to achieve higher predicted accuracy of functional connectivity. To address this issue, we performed a similar experiment mentioned above with two main differences. Firstly, we separately evaluated the inclusion of seven local information indices into the model and compared the prediction accuracy among indices. Secondly, prediction assessment was performed with a reliable and rapid method that avoided vast amounts of calculation and contrastive analysis of network topological properties. The results showed that adding local information to the model allowed good simulations of functional brain network, which reflected its basic characteristics, such as high clustering coefficient, high local efficiency, hub nodes, and small-worldness. Among the local information indices that were tested, “common neighbor” resulted in the best predictions. These results were consistent with the previous research (Vértes et al., 2012), despite using different mathematical models, methods for evaluating topological properties and indices for evaluating network similarity. The current study compares the predictions of functional connectivity with seven local information indices and provides a reference of method selection for construction of prediction models.

## Materials and methods

### Data acquisition and preprocessing

This study was carried out in accordance with the recommendations of the medical ethics committee of Shanxi Province (reference number: 2012013) with written informed consent from all subjects. All subjects have been given written informed consent in accordance with the Declaration of Helsinki. Twenty-eight healthy right-handed volunteers (13 male; mean age: 26.6 ± 9.4 years, range: 17–51 years) underwent resting-state functional magnetic resonance imaging (fMRI) in a 3T MR scanner (Siemens Trio 3-Tesla scanner, Siemens, Erlangen, Germany). Data collection was completed at the First Hospital of Shanxi Medical University. All scans were performed by radiologists who were familiar with magnetic resonance. During the scan, participants were asked to relax with their eyes closed but not to fall asleep. Each scan consisted of 248 contiguous EPI functional volumes (33 axial slices, repetition time (TR) = 2000 ms, echo time (TE) = 30 ms, thickness/skip = 4/0 mm, field of view (FOV) = 192 × 192 mm, matrix = 64 × 64 mm, flip angle = 90°) and the first 10 volumes of time series were discarded regarding magnetization stabilization. See Supplemental Text S1 for detail scanning parameters.

Data preprocessing was performed with SPM8 (http://www.fil.ion.ucl.ac.uk/spm). First, slice-timing correction and head-movement correction were carried out. Two samples exhibiting more than 3.0 mm of translation and 3.0° of rotation were discarded which were not included in the final 28 samples. The corrected images were optimized with a 12-dimensional affine transformation and normalized to 3 × 3 × 3 mm voxel in the Montreal Neurological Institute (MNI) standard space. Finally, linearly detrending and band-pass filtering (0.01–0.10 Hz) were performed to reduce the effects of low-frequency drift and high frequency physiological noise.

### Network construction

An automated anatomical labeling atlas was used to define network nodes (Tzourio-Mazoyer et al., 2002). The whole brain was divided into 90 regions (45 in each hemisphere) and each region was defined as a node in the network. Each regional mean time-series was regressed against the average cerebral spinal fluid (CSF) and white matter signals as well as the six parameters from motion correction. The residuals of these regressions constituted the set of regional mean time-series used for undirected graph analysis. Pearson correlation coefficients among all node pairs in the network were calculated to generate a 90 × 90 correlation matrix. According to predefined thresholds, the correlation matrix was converted into a binary matrix. See supplemental Text S2 for a detailed mathematical definition of the Pearson correlation coefficient.

In the contrast analysis of the complex networks, the compared networks must have the same number of nodes and edges (Bollobás, 2001). Because the quantitative values of topological metrics will depend on size and connection density of the graphs. In order to identify topological differences between graphs pointed to the difference between groups, it is important to control these general effects before making any quantitative comparisons. Sparsity (*S*) was chosen as the threshold to control the number of edges in the networks. *S* was defined as the ratio of real existing edges to the maximum possible number of existing edges. We set the threshold space to be *S*ϵ*[5%, 40%]* because this is the standard used in similar studies (Bullmore and Bassett, 2011) and assures the small-worldness of network, which is one of the most important features in human functional brain networks (Bullmore and Sporns, 2009). Considering the high computing costs, we set the interval in threshold space to 5%. Supplemental Figure S2 illustrates the small-world scalar as a function of sparsity.

### Prediction model mathematic definition

Anatomical distance and nodal local information indices were chosen as parameters to define the mathematical model. Euclidean distance was chosen to define the distance between two given ROIs. Although Euclidean distance between nodal centroids is an imperfect approximation of the anatomical distance between the regions, it has previously been shown to be comparable to more refined diffusion imaging-based measures of connection distance (Supekar et al., 2009).

**Figure 1:**
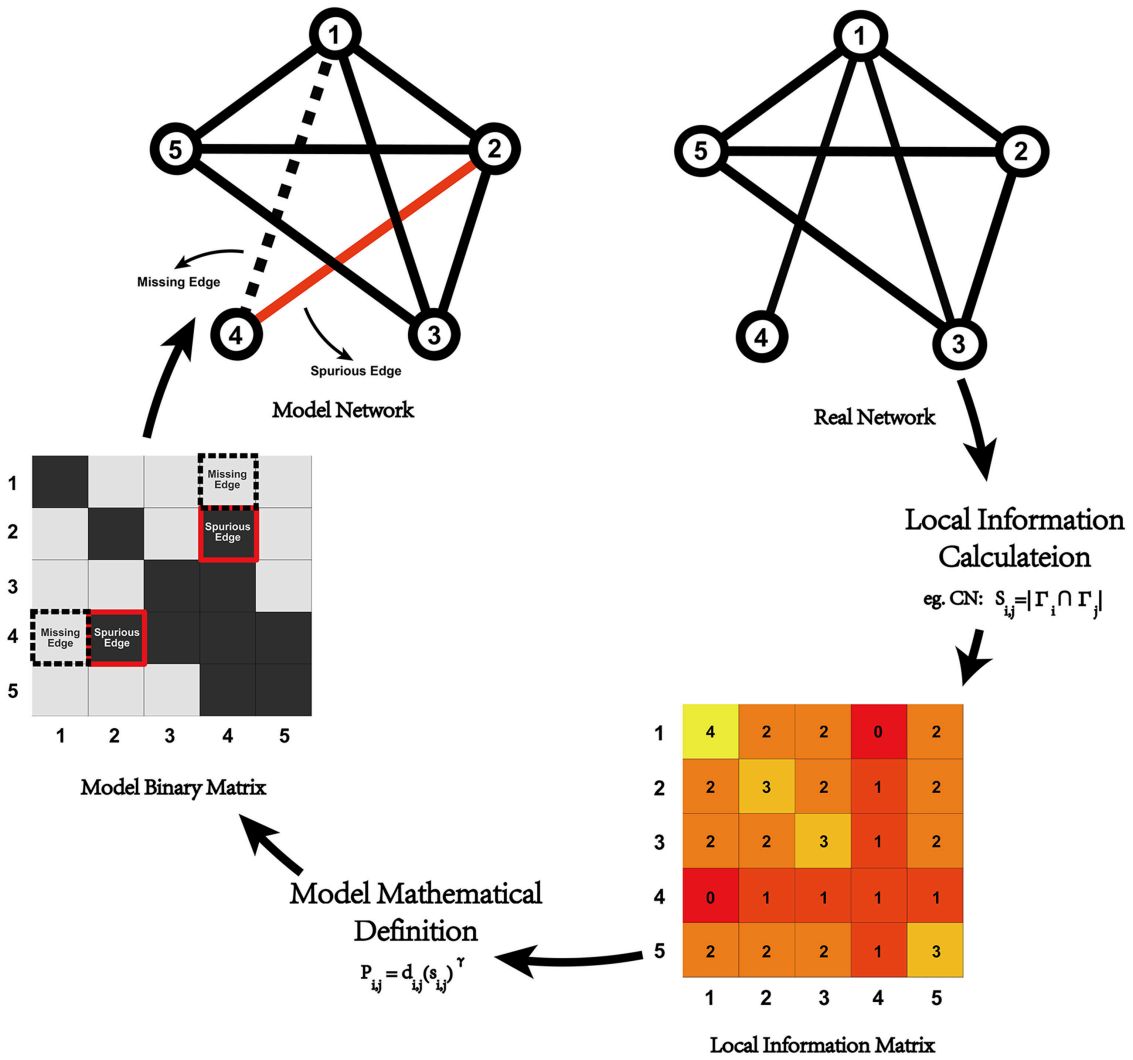
**Illustration of network modeling process with local information indices**. For a given real network, we could calculate the local information between all of the node pairs and generate the corresponding local information matrix (e.g., common neighbor in the illustration), which could be plugged into the model's mathematical definition to generate a predicted network. Compared with real data, some missing and spurious edges might exist, which could lead to some changes in the network's topological properties. In previous research, differences in the topological properties between real data and the predicted networks are always used to quantitatively evaluate the model-predicted effect.

Considering the positive influence that similar topology between nodes has on connections, the introduced nodal local information indices were those used to measure similarity between nodes in complex networks (Lü and Zhou, 2011). The mathematical definition of the prediction model was:

$$P_{i,\;j}=d_{i,\;j}{\left(s_{i,\;j}\right)}^{\gamma },$$

where *P*_*i, j*_ is the probability that a connection between node *i* and node *j* exists, *d*_*i, j*_ is the anatomical distance (Euclidean distance) between node *i* and node *j, s*_*i, j*_ represents the local information indices (seven of which were chosen in the current study), and γ is a constant parameter. Considering computing costs, γ was set to [0, 3], with a step length of 0.1. The modeling process is illustrated in Figure 1.

### Local information indices

The local information index termed “common neighbors” was used for resting-state functional connectivity prediction in a previous study (Vértes et al., 2012). “Common neighbors” is defined as the number of common direct neighbors (nodes that have edges with both nodes *x* and *y*) between the given nodes *x* and *y*. The underlying assumption is that the more common their direct neighbors are, the more similar the topology of a node pair will be and the more likely an edge will be between them. In addition to “common neighbors,” there are many other local information indices. Different indices describe similarity in network nodal topology with different perspectives. We chose to assess seven indices for their usefulness in functional connectivity prediction, including common neighbors (CN) (Liben-Nowell and Kleinberg, 2007), hub depressed index (HDI) (Ravasz et al., 2002), hub promoted index (HPI) (Ravasz et al., 2002), Leicht-Holme-Newman index (LHN-I) (Leicht et al., 2006), Sørensen index (SI) (Sørensen, 1948), preferential attachment index (PA) (Barabasi and Albert, 1999) and resource allocation index (RA) (Zhou et al., 2009). These indices were incorporated into the model's mathematical formula to generate seven functional connectivity prediction models (Table 1). See Supplemental Text S3 for detailed mathematical definitions of the indices. An illustration of prediction networks based on different local information indices is shown in Supplemental Figure S1.

**Table 1:** **Local information indices in the current study**.

**Local information index**	**Index abbreviation**	**Mathematical definition**	**Description**
Common Neighbors (Liben-Nowell and Kleinberg, 2007)	CN	*s*_*i, j*_ = |Γ_*i*_∩Γ_*j*_|	The number of neighbors that x and y have in common.
Hub Depressed Index (Ravasz et al., 2002)	HDI	$s_{i,\;j}=\frac{\left|\Gamma_{i}\cap \Gamma_{j}\right|}{\max\left[k_{i},\;k_{j}\right]}$	Deformation indices of CN. The links adjacent to hubs are probably assigned high scores since the denominator is determined by the higher degree only.
Hub Promoted Index (Ravasz et al., 2002)	HPI	$s_{i,\;j}=\frac{\left|\Gamma_{i}\cap \Gamma_{j}\right|}{\min\left[k_{i},\;k_{j}\right]}$	Deformation indices of CN. An opposite index to HDI.
Leicht-Holme-Newman Index (Leicht et al., 2006)	LHN-I	$s_{i,\;j}=\frac{\left|\underset{i}{\Gamma }\cap \Gamma_{j}\right|}{k_{i}\;\times \;k_{j}}$	Deformation indices of CN. This index gives high similarity to node pairs that have many common neighbors compared not to the possible maximum, but to the expected number of such neighbors.
Preferential Attachment (Sørensen, 1948)	PA	*s*_*i, j*_ = *k*_*i*_ × *k*_*j*_	An index can be used to generate evolving scale-free networks. The probability this new link is connecting *x* and *y* is proportional to *k*_*i*_ × *k*_*j*_.
Resource Allocation (Barabasi and Albert, 1999)	RA	$s_{i,\;j}=\sum_{z\in \Gamma_{i}\cap \Gamma_{j}}\frac{1}{k_{z}}$	This index is motivated by the resource allocation dynamics on complex networks. The similarity between *x* and *y* can be defined as the amount of resource *y* received from *x*.
Sørensen Index (Zhou et al., 2009)	SI	$s_{i,\;j}=\frac{2|\Gamma_{i}\cap \Gamma_{j}|}{k_{i}\;+\;k_{j}}$	Deformation indices of CN. This index is used mainly for ecological community data.

*In the above formulas, S_i, j_ is the local information between node i and j, Γ_i_ is the direct neighbors of node i, k_i_ is the degree of node i, and node z is the common neighbor of node i and j. See Supplemental Text S3 for a detailed mathematical explanation of the above indices*.

### Network topology properties

Eight common global topological properties were chosen for prediction: assortativity, clustering coefficient, characteristic path length, degree distribution, global efficiency, local efficiency, modularity, and transitivity (Table 2). Each property was plotted and the area under the curve (AUC) was calculated. This provided a summarized scalar for the selected threshold space and was a widely used technique in similar studies (Achard and Bullmore, 2007). Multiple linear regression analyses were applied to remove the confounding effects of age, gender and educational attainments for each network properties excluded degree distribution (different from other topological properties, degree distribution shows a distribution function) (independent variable: the AUC of each network properties; dependent variables: age, gender, and educational attainments). The result showed the significant correlation had not been found between network properties and confounding variables (Table 3).

**Table 2:** **Network topological properties in the current study**.

**Property**	**Symbol**	**Description**
Assortativity	*R*	A measure of the correlation between the degree of a node and the mean degree of its nearest neighbors (Newman, 2002).
Clustering Coefficient	*C*	The ratio of the actual number of edges between direct neighbor nodes of given nodes to the number of maximum possible edges for those nodes (Newman, 2003).
Characteristic Path Length	*L*	The average of the shortest path lengths from a given node to other nodes in the network (Newman, 2003).
Degree Distribution	*P(k)*	Frequency distribution of nodal degree in the network (Amaral et al., 2000).
Global Efficiency	*E_*glob*_*	The efficiency of information transmission in the whole network (Latora and Marchiori, 2001).
Local Efficiency	*E_*loc*_*	The efficiency of information transmission from each node to the adjacent nodes (Latora and Marchiori, 2001).
Modularity	*Q*	The degree to which the network may be subdivided into clearly delineated and non-overlapping groups (Newman and Girvan, 2004).
Transitivity	*T*	A variant of the clustering coefficient (Newman, 2003).

**Table 3:** **Results of multiple linear regression analysis between network properties and confounding variables**.

**Confounding variables**	**Coefficients**	**Std. Error**	**T Stat**.	***P***	**Lower 95%**	**Upper 95%**
**ASSORTATIVITY (Adj. R_sqr_ = −0.579, *P* = 0.681)**						
Intercept	0.100	0.087	1.145	0.263	−0.080	0.281
Gender	0.026	0.029	0.876	0.389	−0.035	0.088
Age	0.000	0.001	0.466	0.645	−0.002	0.004
Educational Attainments	0.005	0.013	0.383	0.704	−0.022	0.032
**CLUSTERING COEFFICIENT (Adj. R_sqr_ = 0.004, *P* = 0.391)**						
Intercept	0.419	0.014	29.243	<0.001	0.389	0.448
Gender	0.007	0.004	1.472	0.153	−0.002	0.017
Age	0.000	0.000	−1.119	0.274	0.000	0.000
Educational Attainments	0.001	0.002	0.532	0.599	−0.003	0.005
**CHARACTERISTIC PATH LENGTH (Adj. R_sqr_ = 0.064, *P* = 0.629)**						
Intercept	0.647	0.065	25.331	<0.001	0.513	0.781
Gender	0.001	0.022	1.890	0.196	−0.003	0.087
Age	0.026	0.029	0.876	0.389	−0.035	0.088
Educational Attainments	0.000	0.009	−0.049	0.961	−0.021	0.020
**GLOBAL EFFICIENCY (Adj. R_sqr_ = 0.074, *P* = 0.187)**						
Intercept	0.383	0.015	24.332	<0.001	0.351	0.416
Gender	−0.008	0.005	−1.651	0.111	−0.019	0.002
Age	0.000	0.000	−0.915	0.369	0.000	0.000
Educational Attainments	−0.001	0.002	−0.518	0.608	−0.006	0.003
**LOCAL EFFICIENCY (Adj. R_sqr_ = −0.046, *P* = 0.623)**						
Intercept	0.535	0.018	28.857	<0.001	0.497	0.574
Gender	0.001	0.006	0.213	0.832	−0.011	0.014
Age	0.000	0.000	1.890	0.196	−0.001	0.000
Educational Attainments	0.000	0.002	−0.062	0.951	−0.006	0.005
**MODULARITY (Adj. R_sqr_ = −0.052, *P* = 0.650)**						
Intercept	0.279	0.038	7.341	<0.001	0.201	0.358
Gender	−0.004	0.013	−0.323	0.749	−0.031	0.022
Age	0.000	0.000	−0.388	0.701	−0.001	0.001
Educational Attainments	−0.006	0.005	−1.10	0.278	−0.018	0.005
**TRANSITIVITY (Adj. R_sqr_ = 0.022, *P* = 0.327)**						
Intercept	0.393	0.033	11.672	<0.001	0.323	0.463
Gender	0.018	0.011	1.592	0.124	0.000	0.042
Age	0.000	0.000	0.549	0.587	0.000	0.001
Educational Attainments	0.000	0.005	0.087	0.930	−0.010	0.011

*The range of age is 17–51 years. Optional values of gender are male and female. Optional values of educational attainments are illiteracy, primary school, junior high school, senior high school, junior college, college, graduate degree, and above. Adj. R_sqr_, adjusted R square; Coefficients, regression coefficient; Std. Error, standard error; T stat., T statistic; Lower 95%, low bound of 95% confidence limits. Upper 95%, upper bound of 95% confidence limits*.

### Evaluation of the prediction model

To measure the statistical significance of the prediction, we compared the predicted topological properties with the real data using a two-sample paired nonparametric test, which was corrected with Benjamini and Hochberg false-discovery rate (FDR) method (*q* = 0.05) (Benjamini and Hochberg, 1995). False-discovery rate method, retaining strong control over type 1 error in the context of multiple comparisons, was considered appropriate to correct the small number of comparisons entailed by testing the whole 28 subjects. As has been done in similar EEG (Astolfi et al., 2004), MEG (Fasoula et al., 2013), and structural MRI (Jovicich et al., 2006) studies, we computed the relative error (Guimerà and Sales-Pardo, 2009) to quantitatively evaluate the between-group differences. Relative error was defined as:

$$re=\left|\frac{\left(p_{d}\;-\;p_{m}\right)}{p_{d}}\right|\times 100\%,$$

where *p*_*d*_ is the property value of the real network and *p*_*m*_ is the property value of the predicted network. The evaluation of degree distribution is special. Different from other topological properties, degree distribution shows a distribution function. In the current study, the predicted networks and the real data exhibited an exponential truncated power-law distribution, but the differences were reflected in two parameters: the estimated exponent and the cutoff degree. To quantitatively evaluate the differences between degree distribution functions, the average relative error of the two parameters was computed. The average relative error was defined as:

$$re_{P(k)}=\frac{re_{\alpha }+re_{k_{c}}}{2}\times 100\%,$$

where *re*_α_ is the relative error of the estimated exponent and *re*_*k*_*c*__ is the relative error of the cutoff degree.

To measure the similarity between two networks, we comprehensively considered several network topological properties and defined the network of similar indices, energy *E*, to evaluate the outcome of connectivity prediction. The definition of the *E*-value used here did not consider the weight of the properties. Thus, all of the network's topology properties had equal importance in the model. Energy was defined as:

$$E=\;\frac{1}{re_{R}+re_{C}+re_{L}+re_{E_{loc}}+re_{E_{glob}}+re_{Q}+re_{T}+re_{P(k)}},$$

where *re*_*R*_ is the relative error in assortativity, *re*_*C*_ is the relative error in the clustering coefficient, *re*_*L*_ is the relative error in the characteristic path length, *re*_*E*_*loc*__ is the relative error in local efficiency, *re*_*E*_*glob*__ is the relative error in global efficiency, *re*_*Q*_ is the relative error in modularity, *re*_*T*_ is the relative error in transitivity, and *re*_*P*(*k*)_ is the relative error in degree distribution.

Faced with numerous local information indices, we needed a reliable and rapid prediction evaluation method in order to avoid a large amount of calculation and contrastive analyses of the topological properties. We hypothesized that the more covered the edges were between predicted networks and real data, the better the prediction would be. To test the hypothesis, prediction power was proposed as a metric and a correlation analysis was performed between the *E*-value and the prediction power. In similar studies, prediction power is often used to evaluate link-prediction effects (Cannistraci et al., 2013). Higher prediction power indicates a better prediction, while the closer the prediction power is to 0, the more random the prediction is. Prediction power is defined as:

$$\text{Prediction}\;\text{ Power}=10\;\times \log_{10}\frac{{\text{Pre}}_{\text{M}}}{{\text{Pre}}_{\text{R}}},$$

where Pre_M_ is the ratio of the number of correct edges in a prediction network model to the number of existing edges in real data, and Pre_R_ is the ratio of the number of correct edges in a network model using random prediction methods to the number of existing edges in real data. See Supplemental Text S4 for a detailed mathematical definition and explanation of prediction power.

## Results

**Figure 2:**
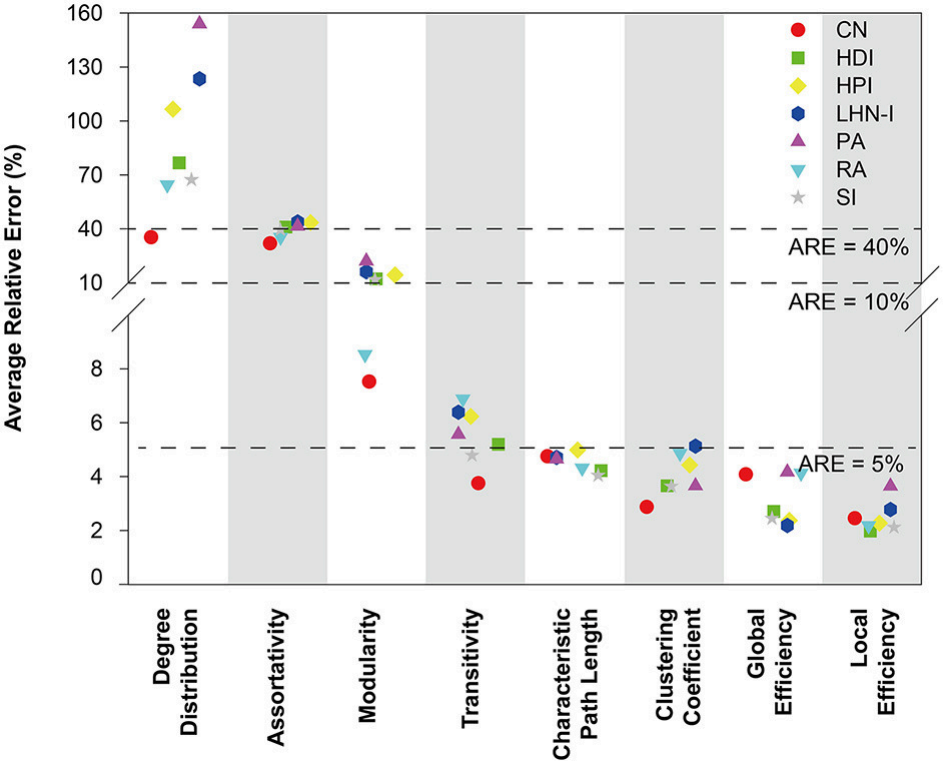
**Average relative error of seven selected local information indices for different topological properties**. The mathematical definition of relative error is $|\frac{\left(p_{\text{d}}-p_{\text{m}}\right)}{p_{\text{d}}}|\times 100\%$. Topological properties were sorted by average relative error. The results showed that the average relative errors of four properties were around or below 5%. CN, common neighbor; HDI, hub depressed index; HDI, hub promoted index; LHN-I, Leicht-Holme-Newman index; SI, Sørensen index; PA, preferential attachment index; RA, resource allocation index.

The average relative error for each network topological property was used to evaluate the predictions. Analysis showed that the different prediction models had different performances in terms of simulating topological properties and most of the predicted network properties were close to the real data (Figure 2). There are four topological properties whose average relative error is less than 5%, including characteristic path length, clustering coefficient, global efficiency, and local efficiency. Modularity and transitivity had relative errors that ranged from 5 to 10%, while assortativity and degree distribution had average relative errors around 40%. Thus, most of the predicted global network properties were close to the real data, except for assortativity and degree distribution. See Supplemental Figure S3 for the detailed information about the distribution of relative errors for each topological property.

**Figure 3:**
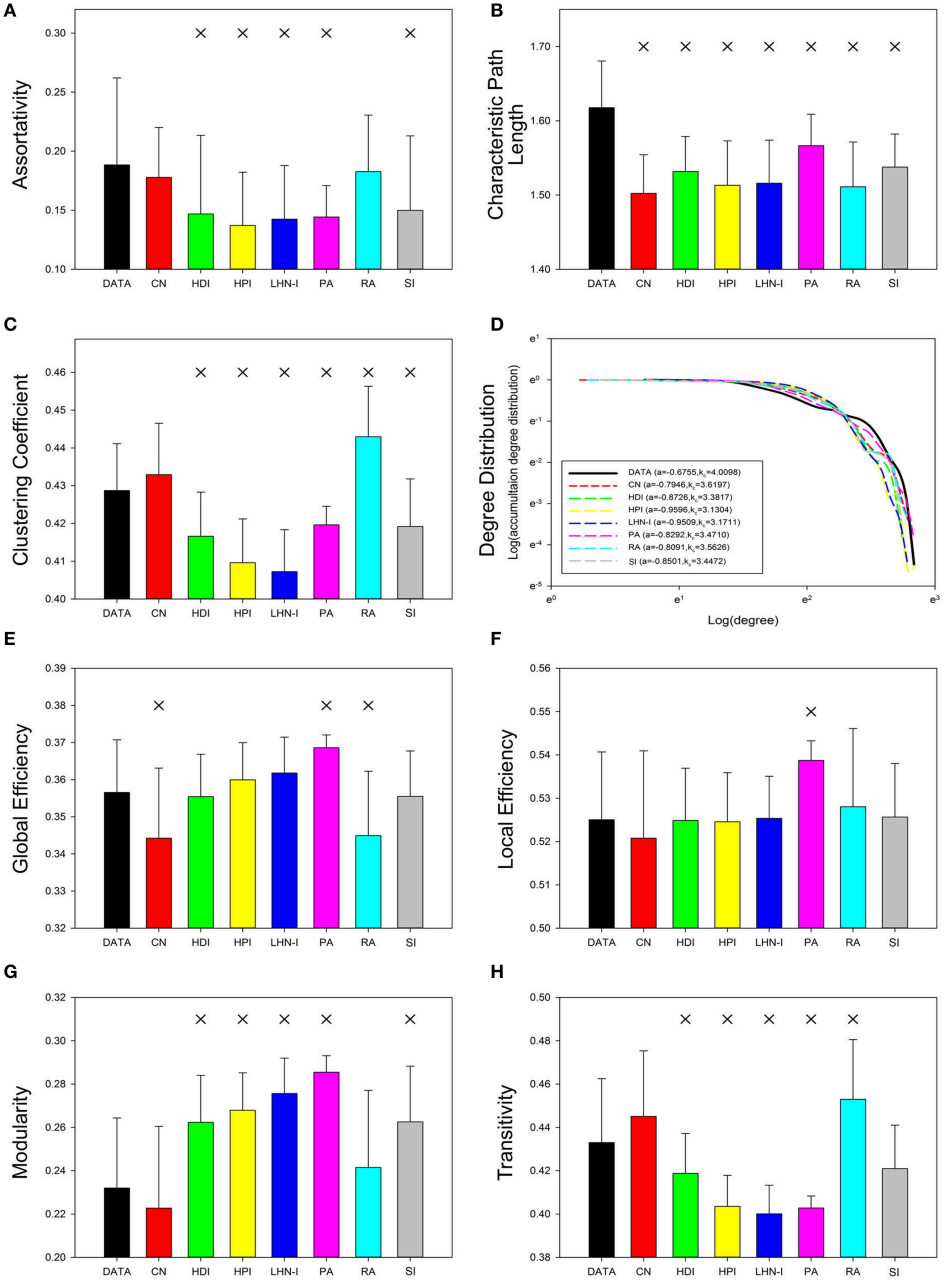
**Topological properties of real data and predicted networks**. Error bars show standard deviation. Asterisks indicate a significant difference between real data and the predicted networks (*p* < 0.05, FDR corrected for 7 comparisons). The statistical test method was a two-sample paired nonparametric test and the corrected method was Benjamini and Hochberg false-discovery rate method (*q* = 0.05). The illustration of degree distribution was on the sparsity of 15%. CN, common neighbor; HDI, hub depressed index; HDI, hub promoted index; LHN-I, Leicht-Holme-Newman index; SI, Sørensen index; PA, preferential attachment index; RA, resource allocation index.

Relative error can be used to quantitatively measure how different predicted networks are from real data. To determine if there were any statistically significant between-group differences, we performed a two-sample paired, nonparametric test with false-discovery rate correction (*q* = 0.05; degree distribution was not statistically analyzed because of its specificity). The results showed that properties with high relative error were always significantly different and low relative error did not necessarily indicate a lack of significant differences. Properties with high relative error, such as assortativity (Figure 3A) and modularity (Figure 3G), showed significant differences in most of the models (*P* < 0.05, FDR corrected for 7 comparisons), except for the CN and RA models. Properties with low relative error, such as characteristic path length (Figure 3B), clustering coefficient (Figure 3C), global efficiency (Figure 3E), local global (Figure 3F), and transitivity (Figure 3H), showed significant differences in some of the models. Degree distribution was not analyzed because of its particularity (Figure 3D). These results suggest that although the property values of the predicted networks were close to those of the real data, the tiny differences were significant.

**Figure 4:**
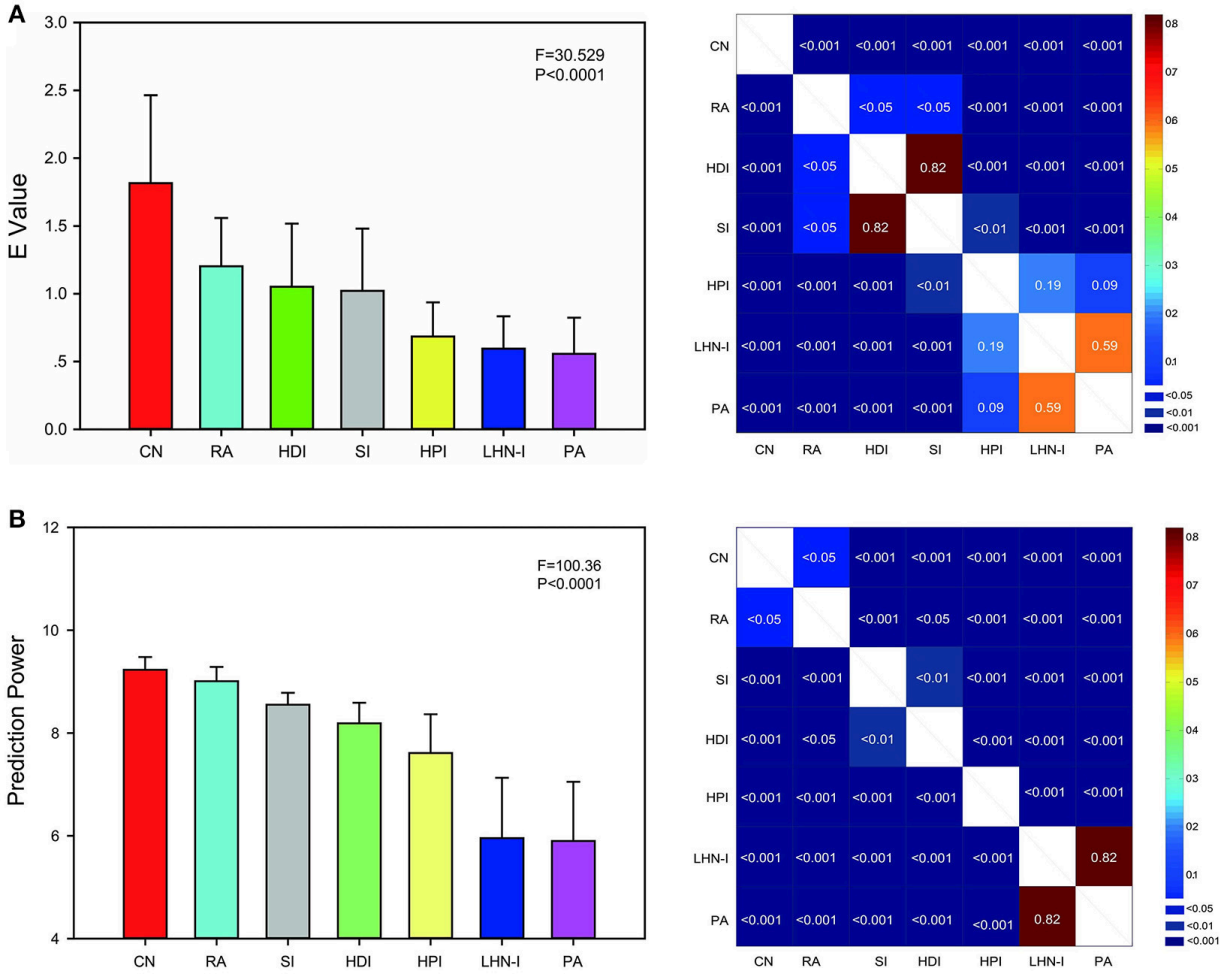
***E* value and prediction power comparison among local information indices. (A)** The *E*-value was used to evaluate the predictions comprehensively. Local information indices were sorted by *E*-value. **(B)** Prediction power was a rapid evaluation metric. Local information indices were sorted by prediction power. Both the evaluation methods showed the similar results that common neighbor showed the best predicted effect and preferential attachment index showed the worst in seven selected local information indexes. Error bars show standard deviation. The *F*-value and *P*-value was the result of one-way ANOVA analysis (uncorrected). The right illustration was the *P*-value of two-sample paired *T*-test between any two indices (FDR corrected for 21 comparisons, *q* = 0.05). CN, common neighbor; HDI, hub depressed index; HDI, hub promoted index; LHN-I, Leicht-Holme-Newman index; SI, Sørensen index; PA, preferential attachment index; RA, resource allocation index.

To comprehensively evaluate the predictions of all the network topological properties in the seven models, we defined a unified measurement metric termed energy (*E*). The *E*-value considered all eight topological property differences. The higher the *E*-value is, the more similar the predicted networks and real data are. The result of ANOVA analysis showed that there were significant differences among seven models (*F* = 30.529, *P* < 0.0001, uncorrected). The results showed that the performance for the CN model was the best among the seven models, with the RA model being second best, and the PA model being the worst (Figure 4A).

Here, we proposed that prediction power could be used to rapidly evaluate the predictions immediately after model generation instead of requiring large computing costs and contrast analysis of model properties. We compared the predictions among seven local information indices by prediction power as well. Significant differences also has been found among seven models (*F* = 100.36, *P* < 0.0001, uncorrected) after ANOVA analysis. The result was very similar to the E value. Both the evaluation metrics showed the similar results. CN showed the best predicted effect and PA showed the worst (Figure 4B). The only change is that SI showed better performance than HDI by prediction power.

**Figure 5:**
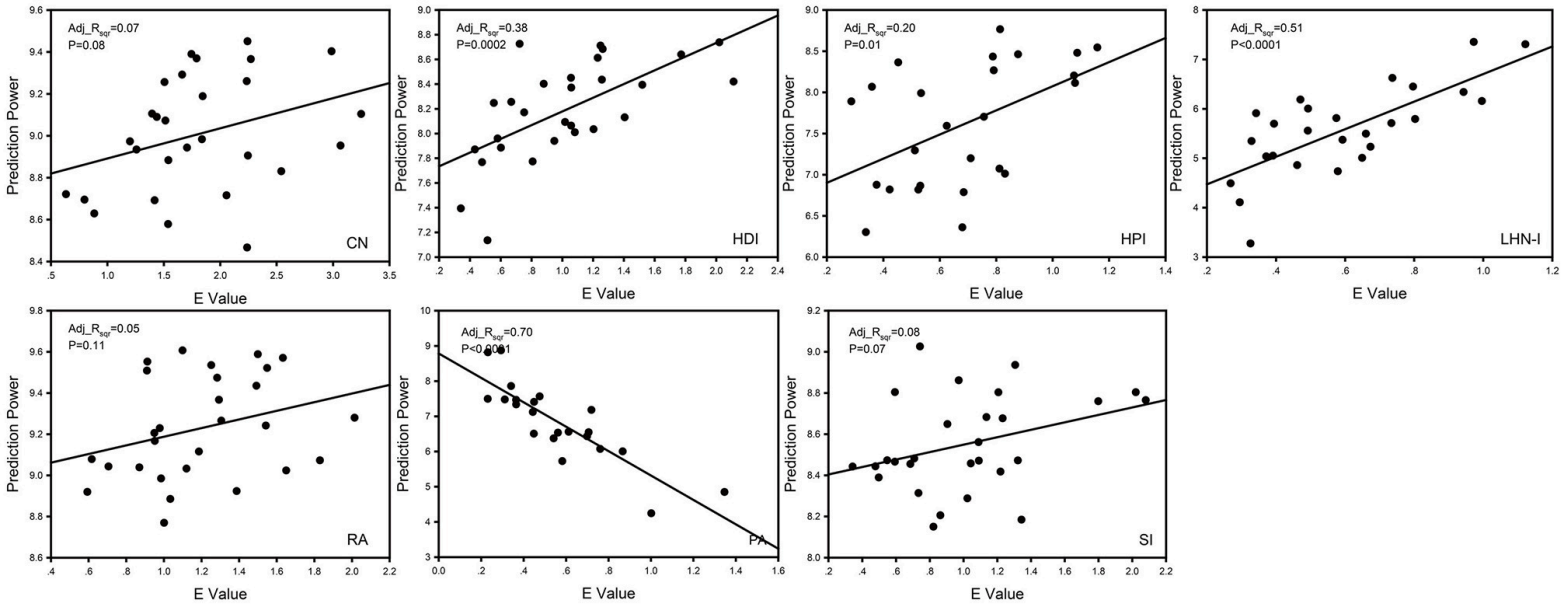
**The correlation analysis between *E*-value and prediction power**. Prediction power was used to evaluate predicted effect rapidly, whose mathematical definition was $10\times \log_{10}\frac{\text{Pr}{\text{e}}_{\text{M}}}{\text{Pr}{\text{e}}_{\text{R}}}$. The results showed that there was a strong correlation between E value and prediction power. Adj_R_sqr_, adjusted R square; CN, common neighbor; HDI, hub depressed index; HDI, hub promoted index; LHN-I, Leicht-Holme-Newman index; SI, Sørensen index; PA, preferential attachment index; RA, resource allocation index.

We performed a correlation analysis between prediction power and *E* value and corrected using the Benjamini & Hochberg false-discovery rate method (*q* = 0.05) (Figure 5; Benjamini and Hochberg, 1995). The four models that showed a significant positive correlation (*p* < 0.05, FDR corrected for 7 comparisons) were HDI, HPI, and LHN-I. The CN and Sorensen models showed a marginally significant correlation (0.05 < *p* < 0.10, FDR corrected for 7 comparisons). Notably, completely different from the other models, the PA model showed a significantly negative correlation (*p* < 0.0001, FDR corrected for 7 comparisons).

## Discussion

As a characteristic of network topology, we proposed local information as a fitting parameter for the predicted models. Local information characterizes network topology and reflects network's local similarity. Our research was able to predict the existence of connections in the brain functional network with local information. The results showed that local information improved the accuracy of predictions. Among the eight network topology properties, most showed good fitting: the relative errors of six properties (characteristic path length, clustering coefficient, global efficiency, local efficiency, modularity, and transitivity) were less than 10%. Additionally, as an efficient type of information within network topology, local information might provide strong evidence regarding the mechanisms of network organization as well as a new viewpoint on the understanding and explanation of network organization (Wang et al., 2012; Zhang et al., 2013).

The results of analysis of E value were consistent with that of another study (Vértes et al., 2012), even though the mathematical model, method of topological property evaluation and indices for evaluating network similarity were different. Similar to our results, predictions from that study were best for the CN model and worst for the PA model (see the detailed comparisons in Table 4). The previous study focused on different mathematical definitions of the prediction model, while our current study focused on comparing prediction ability of a single mathematical model that incorporated differing local information indexes.

**Table 4:** **Comparison of simulated effect evaluation between the current research and the previous similar research**.

**Model**	**Local information**	**Abbr**.	**Mathematical definition**	**Topological properties comparison**								**Energy**
				***P(k)***	***R***	***Q***	***T***	***L***	***C***	***E_*glob*_***	***E_*loc*_***
**THE CURRENT RESEARCH**												
$P_{i,\;j}=d_{i,\;j}{\left(s_{i,\;j}\right)}^{\gamma }$	Common Neighbors	CN	*s*_*i, j*_ = |Γ_*i*_∩Γ_*j*_|	0.353	0.318	0.075	0.038	0.048	0.029	0.041	0.025	1.839
	Resource Allocation	RA	$s_{i,\;j}=\sum_{z\in \Gamma_{i}\cap \Gamma_{j}}\frac{1}{k_{z}}$	0.645	0.354	0.085	0.069	0.043	0.049	0.041	0.022	1.212
	Hub Depressed Index	HDI	$s_{i,\;j}=\frac{\left|\Gamma_{i}\cap \Gamma_{j}\right|}{\max\left[k_{i},\;k_{j}\right]}$	0.767	0.410	0.122	0.052	0.042	0.037	0.027	0.020	1.034
	Sørensen Index	SI	$s_{i,\;j}=\frac{2|\Gamma_{i}\cap \Gamma_{j}|}{k_{i}\;+\;k_{j}}$	0.670	0.396	0.118	0.048	0.041	0.036	0.025	0.021	1.026
	Hub Promoted Index	HPI	$s_{i,\;j}=\frac{\left|\Gamma_{i}\cap \Gamma_{j}\right|}{\min\left[k_{i},\;k_{j}\right]}$	1.066	0.435	0.143	0.062	0.050	0.044	0.024	0.023	0.729
	Leicht-Holme-Newman Index	LHN-I	$s_{i,\;j}=\frac{\left|\Gamma_{i}\cap \Gamma_{j}\right|}{k_{i}\;\times \;k_{j}}$	1.235	0.439	0.162	0.064	0.047	0.051	0.022	0.028	0.628
	Preferential Attachment	PA	*s*_*i, j*_ = *k*_*i*_×*k*_*j*_	1.540	0.414	0.221	0.056	0.047	0.037	0.042	0.037	0.534
**THE PREVIOUS SIMILAR RESEARCH (Vértes et al.,** **2012**)												
$P_{i,\;j}=exp{\left(s_{i,\;j}\right)}^{\gamma }{\left(d_{i,\;j}\right)}^{-\eta }$	Common Neighbors	CN	*s*_*i, j*_ = |Γ_*i*_∩Γ_*j*_|	2 × 10^−4^	–	0.10	–	–	0.79	0.74	–	10^5^
$P_{i,\;j}={\left(s_{i,\;j}\right)}^{\gamma }exp\left(-\eta d_{i,\;j}\right)$	Common Neighbors	CN	*s*_*i, j*_ = |Γ_*i*_∩Γ_*j*_|	10^−3^	–	0.24	–	–	0.85	0.02	–	2 × 10^5^
$P_{i,\;j}={\left(s_{i,\;j}\right)}^{\gamma }{\left(d_{i,\;j}\right)}^{-\eta }$	Variant of Hub Promoted Index	Variant of HPI	*s*_*i, j*_ = max[*k*_*i*_, *k*_*j*_]	5.5 × 10^−4^	–	0.05	–	–	0.95	0.08	–	5 × 10^5^
$P_{i,\;j}={\left(s_{i,\;j}\right)}^{\gamma }exp\left(-\eta d_{i,\;j}\right)$	Variant of Hub Promoted Index	Variant of HPI	*s*_*i, j*_ = max[*k*_*i*_, *k*_*j*_]	1.5 × 10^−5^	–	0.33	–	–	0.41	0.88	–	5 × 10^5^
$P_{i,\;j}={\left(s_{i,\;j}\right)}^{\gamma }{\left(d_{i,\;j}\right)}^{-\eta }$	Variant of Sørensen Index	Variant of SI	*s*_*i, j*_ = *k*_*i*_+*k*_*j*_	7 × 10^−6^	–	0.12	–	–	0.27	0.78	–	6 × 10^6^
$P_{i,\;j}={\left(s_{i,\;j}\right)}^{\gamma }exp\left(-\eta d_{i,\;j}\right)$	Variant of Sørensen Index	Variant of SI	*s*_*i, j*_ = *k*_*i*_+*k*_*j*_	10^−4^	–	0.008	–	–	0.007	0.95	–	2 × 10^8^
$P_{i,\;j}={\left(s_{i,\;j}\right)}^{\gamma }exp{\left(d_{i,\;j}\right)}^{-\eta }$	Variant of Hub Depressed Index	Variant of HDI	*s*_*i, j*_ = min[*k*_*i*_, *k*_*j*_]	9 × 10^−6^	–	0.0003	–	–	0.05	0.34	–	2 × 10^10^
$P_{i,\;j}={\left(s_{i,\;j}\right)}^{\gamma }{\left(d_{i,\;j}\right)}^{-\eta }$	Preferential Attachment	PA	*s*_*i, j*_ = *k*_*i*_×*k*_*j*_	3 × 10^−9^	–	0.001	–	–	0.01	0.80	–	3 × 10^13^
$P_{i,\;j}={\left(s_{i,\;j}\right)}^{\gamma }exp\left(-\eta d_{i,\;j}\right)$	Variant of Hub Depressed Index	Variant of HDI	*s*_*i, j*_ = min[*k*_*i*_, *k*_*j*_]	10^−9^	–	0.004	–	–	0.001	0.65	–	2 × 10^14^
$P_{i,\;j}={\left(s_{i,\;j}\right)}^{\gamma }exp\left(-\eta d_{i,\;j}\right)$	Preferential Attachment	PA	*s*_*i, j*_ = *k*_*i*_×*k*_*j*_	1.5 × 10^−11^	–	0.0001	–	–	0.005	0.51	–	2 × 10^17^

*The above two researches define different topological properties evaluation method and network similarity evaluation indices. Results are sorted by energy value in descending order. Topological properties evaluation method is relative error, whose mathematical definition is $re=|\frac{\left(p_{d}-p_{m}\right)}{p_{d}}|\times \text{100}\%$. Lower value indicates the closer properties between real data and simulated networks. Network similarity indices, energy, was defined as $E=\;\frac{\text{1}}{re_{R}\;+\;re_{C}\;+\;re_{L}\;+\;re_{E_{loc}}\;+\;re_{E_{glob}}\;+\;re_{Q}\;+\;re_{T}\;+\;re_{P\left(k\right)}}$. Higher value indicates the more similar between real data and simulated networks. In the previous similar research (Vértes et al., 2012), results are sorted by energy value in ascending order. Topological properties evaluation method is the P-value associated with the t-test for a difference. P-value is higher than 0.05 indicates no significant difference between real data and stimulated networks. Network similar indices is defined as $\;E=\;\frac{\text{1}}{P_{C}\;\times \;P_{E_{glob}}\;\times \;P_{Q}\times P_{p\left(k\right)}}$. Lower value indicates the more similar between real data and simulated networks. P(k), Degree Distribution; R, Assortativity; C, Clustering Coefficient; L, Characteristic Path Length; E_glob_, Global Efficiency; E_loc_, Local Efficiency; Q, Modularity; T, Transitivity; CN, common neighbor; HDI, hub depressed index; HDI, hub promoted index; LHN-I, Leicht-Holme-Newman index; SI, Sørensen index; PA, preferential attachment index; RA, resource allocation index*.

The mathematical definition of CN is the number of neighbors that two locations x and y have in common. Among the seven models, CN model showed the most accurate predictions. Statistical analysis revealed that five properties (assortativity, clustering coefficient, local efficiency, modularity, and transitivity) within the CN-predicted network did not differ significantly from the real data (*P* > 0.05, FDR corrected for seven comparisons). Meanwhile, the *E*-value for CN model was the highest among all models. As a measurement of network local connectivity, better predictions by CN model implies a higher local connected density in the network. This means that a significant triadic closure structure (Liben-Nowell and Kleinberg, 2007; Zhou et al., 2009) exists in resting-state functional brain networks, which might cause the high clustering coefficient and local efficiency that have been found in resting-state functional brain networks in previous studies (see Bullmore and Bassett, 2011 for a review). This conclusion has been demonstrated in network models with similar properties in the real world, including protein–protein interaction networks (Von Mering et al., 2002), US political blog networks (Ackland, 2005), US air-transportation system networks (Batageli and Pajek, 2006) and social collaboration networks (Newman, 2001). On the contrary, two properties are significantly different between real data and CN model, including characteristic path length and global efficiency. Both properties are related to the long-range links in the network. Compared with regular network, the long-range links in a small-world network ensure the lower characteristic path length, the higher global efficiency and the higher information transferring efficiency. CN evaluated the local similarity but was not sensitive to the long-range links.

Aside from the basic CN model, we also tested HDI, HPI, LHN-I, and SI models, which are deformation indices of CN model. These indices and CN were mentioned as neighborhood-based measures. These deformation indices were subjected to the influence of nodal degree (see the mathematical formula in Table 1). Degree heterogeneity was a measure used to quantify the amount of variation or dispersion of degree of all the nodes in a network (Barabasi and Albert, 1999). If nodal degrees tended to be the same, degree heterogeneity would be very small and there would be no obvious difference between these neighborhood-based measures. In the current study, significant differences in prediction accuracy were found between CN and other neighborhood-based measures, which suggest a high degree of heterogeneity in functional brain networks. The high degree of heterogeneity is an important characteristic of power-law degree distribution (Espinosa et al., 2012), which has been found in the resting-state functional brain network in many studies (see Bullmore and Bassett, 2011 for a review).

Preferential attachment (PA) index was calculated with the least information (only the nodal degree) and resulted in the least accurate predictions. PA was first applied to models of growth networks (Barabasi and Albert, 1999; Mitzenmacher, 2004). The basic premise was that the probability that an edge has node x as an endpoint is proportional to the current number of neighbors of x. Similar mechanisms could also lead to scale-free networks without growth (Xie et al., 2008). Therefore, PA resulted in accurate predictions in a scale-free network. However, PA performance was disappointing, compared with other indices in small-world networks, such as human functional brain networks (Vértes et al., 2012) and nervous system networks for other species (Cannistraci et al., 2013). Studies have shown that PA performs badly in a rich-club network consisting of many components (Zhou et al., 2009). The same properties exist in human functional brain networks (van den Heuvel and Sporns, 2011). Our results show that PA is not suitable for predicting functional connectivity.

Unlike other indices, RA focuses on the degree of direct neighbor. Consider a pair of nodes, x and y, which are not directly connected. Node x can send resources to y, with their common neighbors playing the role of transmitters. In the simplest case, we assume that each transmitter has one unit of resource and will distribute it evenly to all its neighbors. The similarity between x and y can be defined as the amount of resource y received from x. Like CN, RA is suitable for networks with a large clustering coefficient, a high degree of heterogeneity (Zhou et al., 2009), in which resources tend to flow to high-degree nodes rather than low-degree nodes (Ou et al., 2007). Functional brain networks have been demonstrated to organize intrinsically as highly modular small-world architectures, capable of transferring information at a low wiring cost efficiently as well as formatting highly connected hub nodes. Hub node is usually defined as the node with a degree greater than the mean degree plus the standard deviation (He et al., 2009). Previous research has shown that brain networks are vulnerable to a targeted attack on hub nodes—expressed in the significant reduction of connectivity and efficiency—regardless of whether the network is structural (He et al., 2007) or functional (Crossley et al., 2014).

Faced with numerous local information indices, we needed a reliable and rapid method to evaluate the prediction accuracy of the models. As a widely used measurement in link-prediction research, prediction power was introduced to evaluate the predictions. Correlation analysis with the *E* value revealed a strong positive correlation. Six of the local information indices showed significant or marginally significant correlations, while PA index appeared more suitable for scale-free networks than for small-world networks. The result fully verified our hypothesis and implied that we could transform the problem of prediction evaluation into a problem of link prediction, which avoids vast amounts of calculation and contrastive analysis of network topological properties.

## Methodology

Different from other methods, local information is a topological property of functional network itself. This means that the topological properties of the network itself are used to predict its own connections. This is circular. The precondition for this thinking is that we have a complete network (all connections in the given network are known). In contrast, if we have an incomplete network because the connection data are difficult to collect or the connection computation costs are high, we can predict the missing connections with the known connections. That is the value of the method.

Local information is a common method of link prediction, which is among the most important research fields in network science. The aim of link prediction is to predict the existence of potentially missing connections and to evaluate the reliability of the existing connections according to the available incomplete or unreliable network. The same problems occur in brain network research. We thus applied link-prediction methods to this field and hoped it could be the part of solution.

Large-scale brain network construction is an intractable and urgent problem. Benefiting from the promotion of hardware performance and advancement of computation frames, brain networks can be constructed at the voxel level. However, at larger scales (e.g., the neuron level), constructing a complete network is difficult. A huge computational cost must be paid to construct a complete network because the numbers of nodes and connections are enormous. In this case, link prediction can solve this problem to some extent, so long as it can provide satisfactory accuracy. Generally, application of link prediction in network construction decreases computation costs at the risk of increasing the error rate.

Apart from predicting missing connections, link prediction can also be used to evaluate the reliability of existing connections in an unreliable network, namely the possibility of pseudo connections. The connections in brain networks also need to be verified reliably, regardless of whether the networks are structural or functional. Previous research has lacked methods of quality control when constructing brain networks, which are disturbed by many factors. How do we know that the connections we obtained through correlation analysis actually exist in the brain? How can we to verify the reliably of the connections in network? There are two key points to solving this problem—a dependable method for quantifiable evaluation and a comparable golden rule. Link prediction is a choice that can satisfy the first point. For a given network, we can evaluate the possibility of a pseudo connection with the link-prediction method. For the latter point, unlike in functional networks, tract-tracing measures provide an importance reference for reconstructing pathways in DTI structural networks (non-human species).

## Conclusion

Anatomical distance has widely been used to predict functional connectivity because of the potential relationship between structural connectivity and functional connectivity. But studies have shown that one-parameter model (anatomical distance) cannot account for the small-worldness, modularity, and degree distribution of normal human brain functional networks.

Local information is the simplest and direct method in link-prediction research, which utilizes relevant network topological information to predict the possibility of an edge between two given nodes in a network. The underlying basic assumption was that the higher similarity between the nodes in network, the higher probability of an edge existing between them. Based on previous researches, the current study separately evaluated the inclusion of seven local information indices into the model and compared the prediction accuracy among indices. Results showed that the simulated networks reflected the basic characteristics of brain networks, such as high clustering coefficient, high local efficiency, hub nods, and small-worldness. But when it comes to some properties related with long-range links, the simulated result is disillusionary. It reflected the limitation of local information method. Local information method evaluated the local similarity well and but it was not sensitive to the long-range links.

As is mentioned in Methodology, the main application of local information is in incomplete or unreliable network. For an incomplete network, in which the existence of some connections is unknown, local information can predict the missing connections. For an unreliable network, in which some existing connections might not be real, local information can evaluate the reliability of connections. Both of them are intractable problems in brain network construction, especially the latter. We think local information method has practical applicability in brain network research as a feasible and effective tool.

## Author contributions

CC was responsible for the study design and writing the manuscript. JC performed data analysis and statistical processing. XC provided and integrated experimental data. HG was the heads of the funds and supervised the paper. All authors approved the final version of the manuscript.

### Conflict of interest statement

The authors declare that the research was conducted in the absence of any commercial or financial relationships that could be construed as a potential conflict of interest.
