# Supplementary material for: Comparison of Local Information Indices Applied in Resting State Functional Brain Network Connectivity Prediction
Source: Front Neurosci. 2016 Dec 27;10:585. doi: 10.3389/fnins.2016.00585 (PMC5186779; doi:10.3389/fnins.2016.00585)
Supplement: Supplementary file 2 [file Presentation2.PDF]

Supplemental Text S2. Mathematical Definition of Pearson Correlation Coefficient

We used Pearson correlation coefficient to calculate the correlation coefficient of average time series between any two nodes. The mathematical definitions follows below:

$$r = \frac{\sum_{i=1}^{n}(X_i - \overline{X})(Y_i - \overline{Y})}{\sqrt{\sum_{i=1}^{n}(X_i - \overline{X})^2}\sqrt{\sum_{i=1}^{n}(Y_i - \overline{Y})^2}}$$

X_i and Y_i respectively represent time series of voxel i and j. We then generated a N × N time series correlation matrix. Here, N is the number of node in the given parcellation.
