# Supplementary material for: Comparison of Local Information Indices Applied in Resting State Functional Brain Network Connectivity Prediction
Source: Front Neurosci. 2016 Dec 27;10:585. doi: 10.3389/fnins.2016.00585 (PMC5186779; doi:10.3389/fnins.2016.00585)
Supplement: Supplementary file 3 [file Presentation3.PDF]

Supplemental Text S3. Mathematical Definition of Local Information Indexes

Common Neighbors

For a node i, let Γ_i denote the set of neighbors of i. In common sense, two nodes, i and j, are more likely to have a link if they have many common neighbors. The simplest measure of this neighborhood overlap is the directed count [1], namely

$$s_{i,j} = |\Gamma_i \cap \Gamma_j|$$

Hub Promoted Index

This index is proposed for quantifying the topological overlap of pairs of substrates in metabolic networks [2], defined as

$$s_{i,j} = \frac{|\Gamma_i \cap \Gamma_j|}{min[k_i, k_j]}$$

Under this measure, the links adjacent to hubs (here, the term "hub" represents node with very large degree) are probably assigned high scores since the denominator is determined by the lower degree only.

Hub Depressed Index

Analogously to the above index, we consider a measure with opposite effect on hubs for comparison [2], which is defined as

$$s_{i,j} = \frac{|\Gamma_i \cap \Gamma_j|}{max[k_i, k_j]}$$

Leicht-Holme-Newman Index

This index gives high similarity to node pairs that have many common neighbors compared not to the possible maximum, but to the expected number of such neighbors [3]. It is defined as

$$s_{i,j} = \frac{|\Gamma_i \cap \Gamma_j|}{k_i \times k_j}$$

where the denominator, $k_i \times k_j$, is proportional to the expected number of common neighbors of nodes i and j in the configuration model [4].

Preferential Attachment

The mechanism of preferential attachment can be used to generate evolving scale-free networks

(i.e., networks with power-law degree distributions), where the probability that a new link is connected to the node i is proportional to k_i [5]. Similar mechanism can also lead to scale-free networks without growth [6], where at each time step, an old link is removed and a new link is generated. The probability this new link is connecting i and j is proportional to $k_i \times k_j$. Motivated by this mechanism, a corresponding similarity index can be defined as

$$s_{i,j} = k_i \times k_j$$

which has already been suggested as a proximity measure [7], as well as been used to quantify the functional significance of links subject to various network-based dynamics, such as percolation [8], synchronization [9] and transportation [10]. Note that, this index requires less information than all others, namely it does not need to know the neighborhood of each node. As a consequence, it also has the minimal computational complexity.

Resource Allocation

Considering a pair of nodes, i and j, which are not directly connected. The node i can send some resource to j, with their common neighbors playing the role of transmitters. In the simplest case, we assume that each transmitter has a unit of resource, and will averagely distribute it to all its neighbors [11]. The similarity between i and j can be defined as the amount of resource j received from i, which is:

$$s_{i,j} = \sum_{z \in \Gamma_i \cap \Gamma_j} \frac{1}{k_z}$$

Sørensen Index

This index is mainly used for ecological community data [12], which is defined as

$$s_{i,j} = \frac{2\left|\Gamma_i \cap \Gamma_j\right|}{k_i + k_j}$$

Where Γ_i denote the set of neighbors of i, k_i denote the degree of the node i.

References

1. Liben‐Nowell, D. and J. Kleinberg, *The link‐prediction problem for social networks.* Journal of the American society for information science and technology, 2007. **58**(7): p.

1019-1031.

2. Ravasz, E., et al., *Hierarchical organization of modularity in metabolic networks.* Science, 2002. **297**(5586): p. 1551-1555.

3. Leicht, E., P. Holme, and M.E. Newman, *Vertex similarity in networks.* Physical Review E, 2006. **73**(2): p. 026120.

4. Molloy, M. and B.A. Reed, *A critical point for random graphs with a given degree sequence.* Random structures and algorithms, 1995. **6**(2/3): p. 161-180.

5. Sørensen, T., *A method of establishing groups of equal amplitude in plant sociology based on similarity of species and its application to analyses of the vegetation on Danish commons.* Biol. Skr., 1948. **5**: p. 1-34.

6. Xie, Y.-B., T. Zhou, and B.-H. Wang, *Scale-free networks without growth.* Physica A: Statistical Mechanics and its Applications, 2008. **387**(7): p. 1683-1688.

7. Huang, Z., X. Li, and H. Chen. *Link prediction approach to collaborative filtering*. in *Proceedings of the 5th ACM/IEEE-CS joint conference on Digital libraries*. 2005. ACM.

8. Holme, P., et al., *Attack vulnerability of complex networks.* Physical Review E, 2002. **65**(5): p. 056109.

9. Yin, C.-Y., et al., *Decoupling process for better synchronizability on scale-free networks.* Physical Review E, 2006. **74**(4): p. 047102.

10. Zhang, G.-Q., D. Wang, and G.-J. Li, *Enhancing the transmission efficiency by edge deletion in scale-free networks.* Physical Review E, 2007. **76**(1): p. 017101.

11. Barabasi, A.L. and R. Albert, *Emergence of scaling in random networks.* Science, 1999. **286**(5439): p. 509-12.

12. Zhou, T., L. Lü, and Y.-C. Zhang, *Predicting missing links via local information.* The European Physical Journal B-Condensed Matter and Complex Systems, 2009. **71**(4): p. 623-630.
