# Supplementary material for: Comparison of Local Information Indices Applied in Resting State Functional Brain Network Connectivity Prediction
Source: Front Neurosci. 2016 Dec 27;10:585. doi: 10.3389/fnins.2016.00585 (PMC5186779; doi:10.3389/fnins.2016.00585)
Supplement: Supplementary file 4 [file Presentation4.PDF]

Supplemental Text S4. Mathematical Definition of Precision and Prediction Power

Precision

Given the ranking of the non-observed links, the Precision is defined as the ratio of relevant items selected to the number of items selected [1]. That is to say, if we take the top-L links as the predicted ones, among which L_r links are right (i.e., there are L_r links in the probe set E_P), then the Precision equals L_r/L. Clearly, higher precision means higher prediction accuracy.

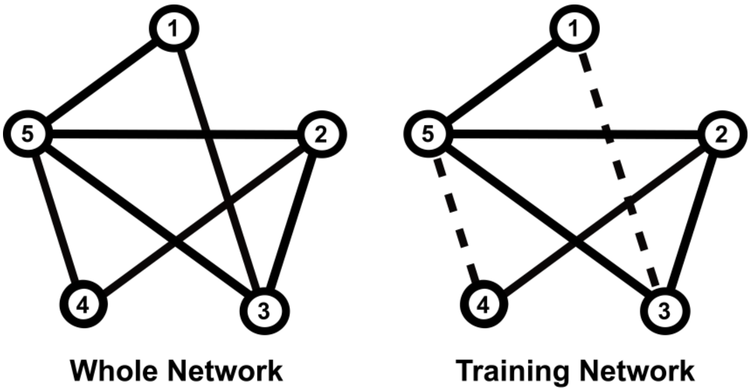

Figure 1. The Illustration of the calculation of Precision

Figure 1 shows an example of how to calculate the Precision. In this simple graph, there are five nodes, seven existent links and three nonexistent links ((1, 2), (1, 4) and (3, 4)). To test the algorithm's accuracy, we need to select some existent links as probe links. For instance, we pick (1, 3) and (4, 5) as probe links, which are presented by dash lines in the right plot. Then, any algorithm can only make use of the information contained in the training graph (presented by solid lines in the right plot). If an algorithm assigns scores of all non-observed links as s12 = 0.4, s13 = 0.5, s14 = 0.6, s34 = 0.5 and s45 = 0.6. To calculate Precision, if L = 2, the predicted links are (1, 4) and (4, 5). Clearly, the former is wrong while the latter is right, and thus the Precision equals 0.5.

Prediction Power

To characterise the deviation of each predictor from randomness, we transformed the indices' mean precisions at each sparsification level into decibels as

$$\text{Prediction Power} = 10 \times \log_{10} \frac{\text{Pre}_\text{M}}{\text{Pre}_\text{R}}$$

taking the mean performance of the random predictor as a reference [2]. Pre_M is the precision of simulation model. Pre_R is the precision of random method. The value of prediction power is more close to 0, the prediction effect is more trended to random prediction. The predictive power, measured at different sparsification levels, generated a prediction power curve, and the area under this curve summarised the power of each predictor.

Reference

1. Schafer, J.B., et al., *Collaborative filtering recommender systems*, in *The adaptive web*2007, Springer. p. 291-324.
2. Cannistraci, C.V., G. Alanis-Lobato, and T. Ravasi, *From link-prediction in brain connectomes and protein interactomes to the local-community-paradigm in complex networks.* Scientific reports, 2013. **3**.
