# Supplementary material for: Comparison of Local Information Indices Applied in Resting State Functional Brain Network Connectivity Prediction
Source: Front Neurosci. 2016 Dec 27;10:585. doi: 10.3389/fnins.2016.00585 (PMC5186779; doi:10.3389/fnins.2016.00585)
Supplement: Supplementary file 5 [file Image1.PDF]

Supplemental Figure S1. Illustration of resting state functional brain network stimulate model based on local information indexes

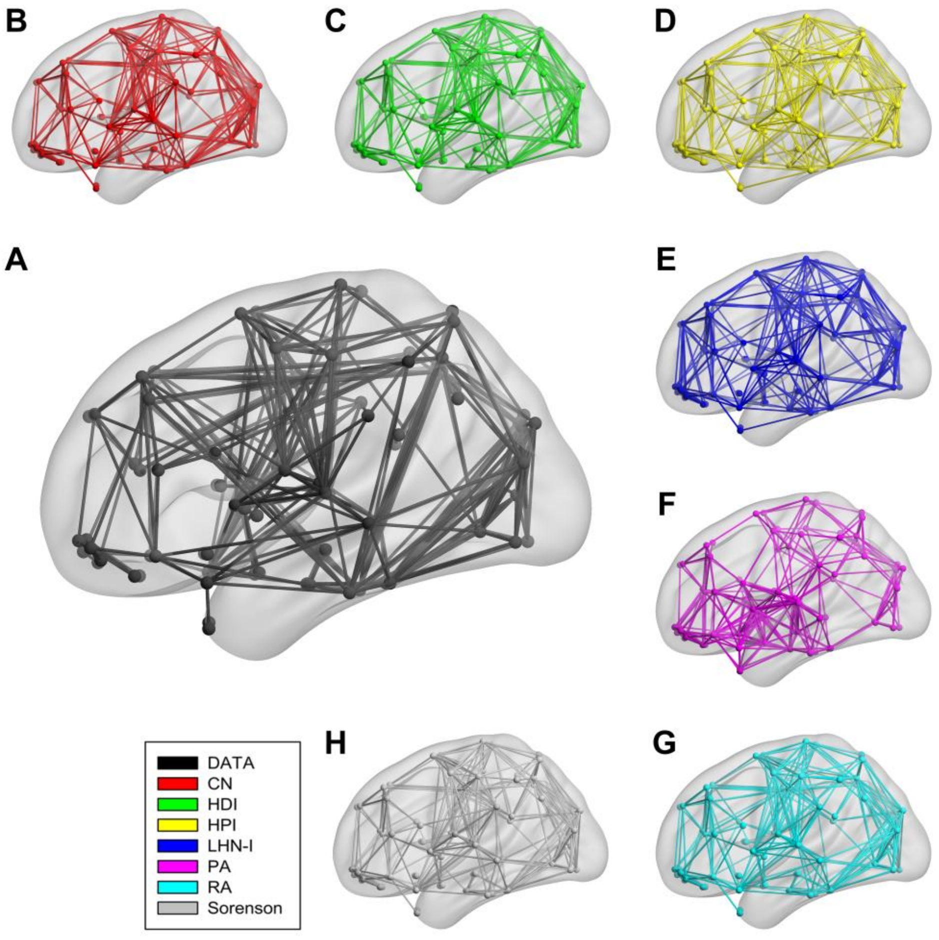

Figure S1. Illustration of resting state functional brain network stimulate model based on local information indexes. Automated anatomical labeling atlas was used to define node and Pearson correlation was used to define edge. In this illustration, network sparsity is 15%. The nodes were mapped onto the cortical surfaces using BrainNet viewer software (http://www.nitrc.org/projects/bnv/). CN, common neighbor; HDI, hub depressed index; HDI, hub promoted index; LHN-I, Leicht-Holme-Newman index; SI, Sørensen index; PA, preferential attachment index; RA, resource allocation index.
