# Supplementary material for: Comparison of Local Information Indices Applied in Resting State Functional Brain Network Connectivity Prediction
Source: Front Neurosci. 2016 Dec 27;10:585. doi: 10.3389/fnins.2016.00585 (PMC5186779; doi:10.3389/fnins.2016.00585)
Supplement: Supplementary file 6 [file Image2.PDF]

Supplemental Figure S2. Illustration of small-world scalar as a function of sparisity

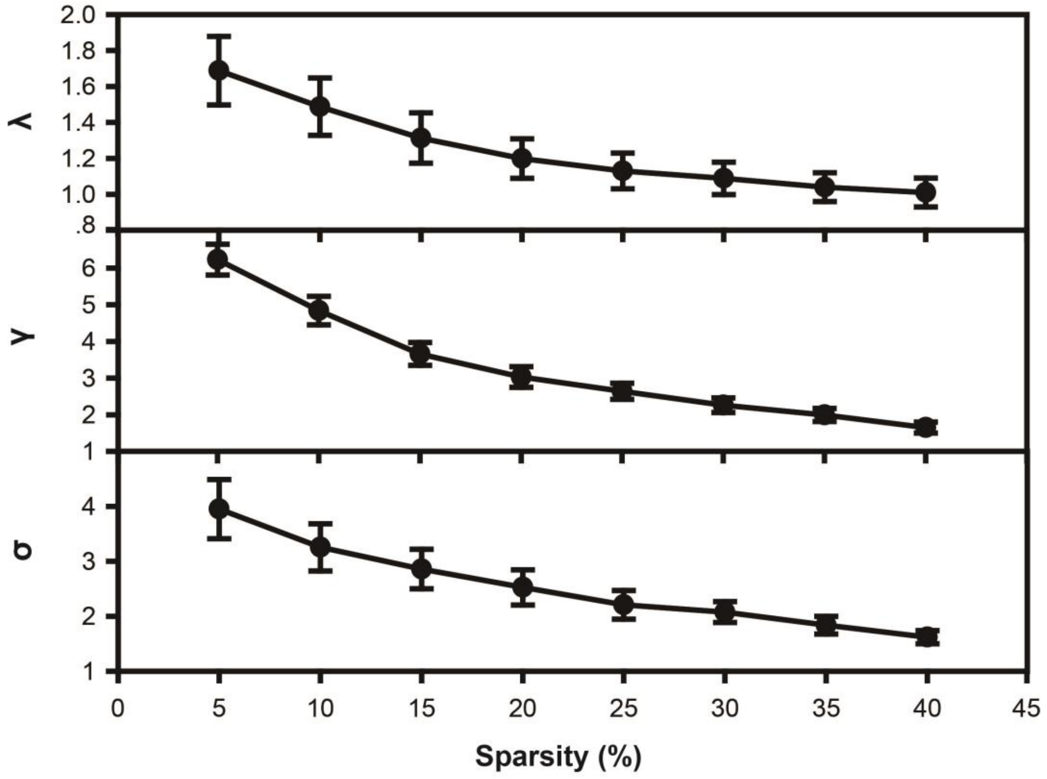

Figure S2. Illustration of small-world scalar as a function of sparisity Error bar is standard deviation. λ, normarlized characteristic path length; γ, normarlized clustering coefficient; σ, small-worldness scalar.
