# Supplementary material for: Comparison of Local Information Indices Applied in Resting State Functional Brain Network Connectivity Prediction
Source: Front Neurosci. 2016 Dec 27;10:585. doi: 10.3389/fnins.2016.00585 (PMC5186779; doi:10.3389/fnins.2016.00585)
Supplement: Supplementary file 7 [file Image3.PDF]

Supplemental Figure S3. Data distribution of the relative error of the topological properties

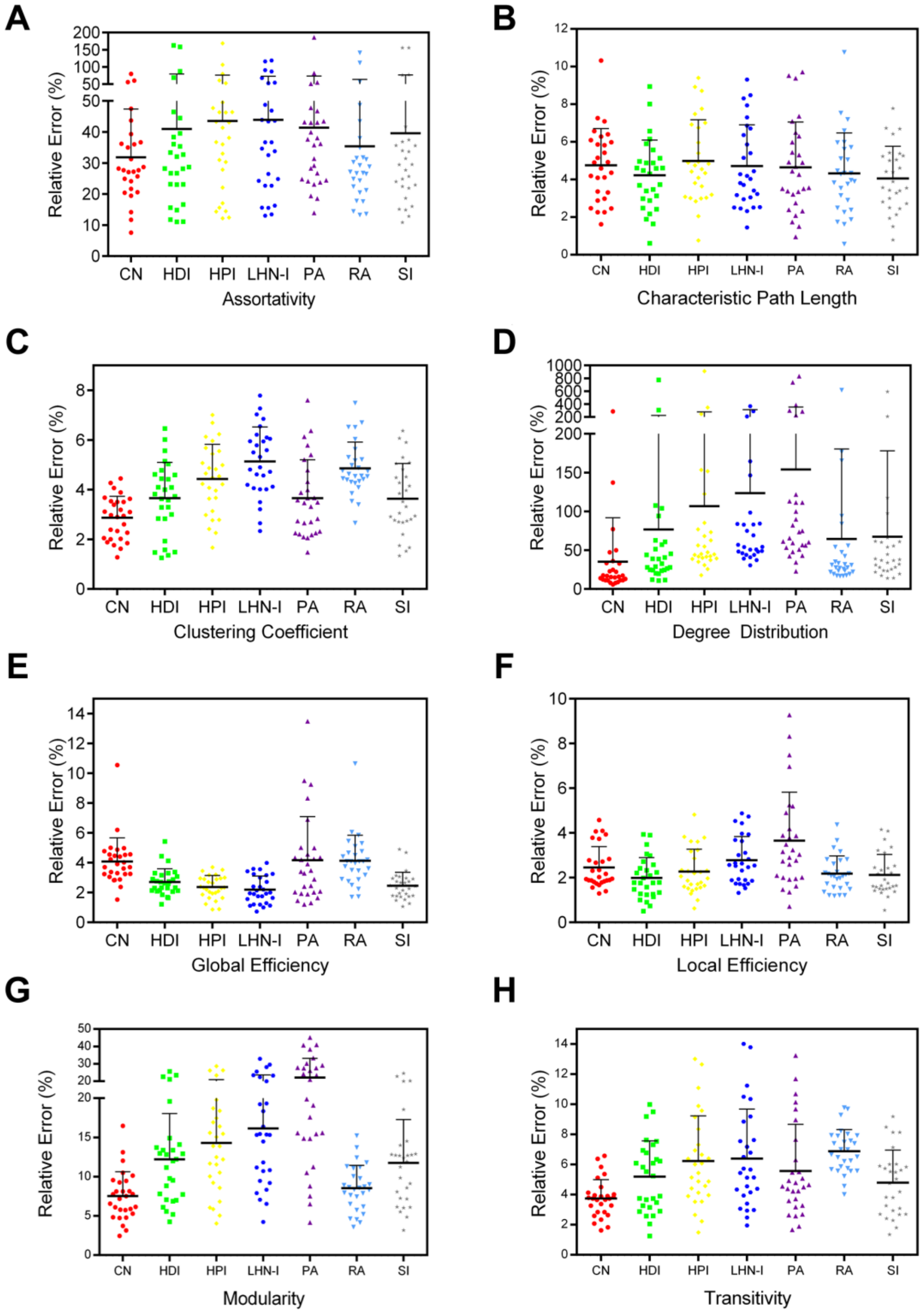

Figure S3. Data distribution of the relative error of the topological properties. Thick horizontal denotes arithmetic mean value. Error bar is standard deviation. CN, common neighbor; HDI, hub depressed index; HDI, hub promoted index; LHN-I, Leicht-Holme-Newman index; SI, Sørensen index; PA, preferential attachment index; RA, resource allocation index.
